# Optimal Analysis of Human Resources Allocation in Colleges and Universities Based on Internet of Things Technology

**DOI:** 10.1155/2021/9605397

**Published:** 2021-12-27

**Authors:** Ai Zhang, Zhengmao Ju, Yiling Liu

**Affiliations:** ^1^Gannan University of Science and Technology, Jiangxi, Ganzhou 341000, China; ^2^College of Applied Sciences, Jiangxi University of Technology, Jiangxi, Ganzhou 341000, China; ^3^Mokwon University, Daejeon 34705, Republic of Korea

## Abstract

This article takes the development and superiority of the human resource system under the IoT technology as the background and adopts the popular IoT architecture and the current latest technical theories to develop and improve the human resource management system. First, we introduce the establishment of the J2EE development environment and then introduce the realization of the three layers of the overall IoT architecture of the system, namely, the realization of the presentation layer, the realization of the layer, and the realization of the data and then the realization of the main modules of the system; finally, we explain the function test of some modules of the system. After that, we mainly analyze the requirements of the system, first the overall requirements analysis and then the function and nonfunctional requirements analysis of the system. On the basis of perfecting the service-oriented modeling of manufacturing resources, the integration and optimization of resources are carried out according to the requirements of manufacturing tasks, and the discovery mechanism and matching methods of manufacturing resource services are studied to reduce the solution space of resource allocation. Aiming at the problem of optimal utilization of manufacturing resources, this paper establishes a scientific and reasonable evaluation index system to reduce resource service costs and improve resource utilization. At the same time, after analyzing the problem of real-time distribution of resources in the manufacturing Internet of Things environment, the real-time information of resource distribution is designed. The interactive mechanism and a two-tier optimization method based on real-time information-driven resource distribution tasks are proposed. The simulation experiment results show that the optimization method proposed in this paper, compared with the traditional resource distribution method, not only reduces the carrying distance of human resource distribution but also reduces the empty load rate, thereby reducing the waste of human resources.

## 1. Introduction

With the help of this technology, traditional human resource management is also facing unprecedented opportunities and challenges [1]. In enterprises, human resource management has become an indispensable part of enterprise management. Manual human resource management is time-consuming and laborious, prone to errors, cumbersome, and inefficient, and the existing personnel system is highly coupled with the enterprise [2]. Therefore, the previous management model has changed and been replaced by the information network management model. With the penetration of the concept of the Internet of Things in the manufacturing field, the development process of manufacturing companies has evolved from the traditional “black box” model to a “three-dimensional space plus time multidimensional, transparent ubiquitous perception” model, which is driven by information perception technology [3–5]. This article uses advanced computer technology to develop enterprise human resource management system, networked and informatized human resource management. Such a system can reduce the work burden of enterprise employees, move employees' positions in real time and efficiently according to local conditions, maximize employees' potential, and improve the economic benefits of the enterprise [6–8].

Li et al. [9] proposed a shift in the focus of corporate personnel information management, which marked the emergence of research on the theory and practical application of human resource management. The human resource management system developed by Oracle mainly reduces the cost of enterprise management through system analysis, management mode, and establishment of employee incentive mechanisms. The human resource management system developed by IBM mainly provides solutions and management services. The recruitment of teachers in space is open, and the teacher data and other employment information of the schools are very transparent, which has achieved a reasonable flow of talents [10–13]. Chen et al. [14] proposed a new service-oriented model of physical entities and proposed a method of secondary combination reasoning to speed up the combination process. In the same year, they further proposed a new context-aware resource-led service model. Awoyemi et al. [15] studied the extended OWL-S model to describe the contextual characteristics of service providers in CPS and also studied technology for generating task demand planning. Fang et al. [16] conducted research on dynamic material distribution in the workshop under the IoT environment. Aiming at the problems of low transparency in the production process, difficulty in collecting material information, low material distribution efficiency, and poor information interaction, they proposed a real-time sensing information-driven. Fischer et al. [17] studied the application of the Internet of Things technology in the disaster relief material distribution management system, using the real-time monitoring data collected by the Internet of Things to analyze and judge the results and play a comprehensive auxiliary decision-making process for the entire material distribution and transportation process. Some scholars studied the material distribution and monitoring system of mixed-flow assembly workshops and put forward the overall framework of material distribution and tracking in response to the requirements of material distribution and monitoring on the production site and assembly error prevention [18]. Since the status information of each distribution carrier, pallet, and so forth (such as location, material category, and quantity) can be sensed in real time, the description of dynamic resources based on real-time process perception can be further optimized. In addition, by constructing a real-time information interaction mechanism and perfecting the optimization of real-time information-driven resource distribution tasks, it is also a problem to be finished in resource distribution in the manufacturing Internet of Things environment [19–21].

In order to cope with the future expansion of the functions of the Internet of Things enterprises, this system adopts J2EE technology, B/S platform mode, and SSH framework to develop the human resource deployment system. Logging in to office is in the concept of cloud office. At the same time, the ULHA evaluation model is introduced into the enterprise human resource management system to better help the enterprise evaluate the company's employees reasonably, conduct comprehensive and humanized management, and help companies make better decisions in the recruitment process. Due to the complexity of the regression model, it cannot be done by relying solely on the spatial analysis function of GIS. The regression analysis must be done with the help of professional statistical analysis software SPSS. Firstly, the background and significance of the system research and the main existing problems are explained, and the related technologies and theories of the system are introduced; secondly, the functional requirements and nonfunctional requirements of the system are analyzed; and finally, the main modules of the system are implemented and tested, and the test results basically meet the design requirements of the system. The developed system is easy to operate, simple to use, and easy to expand, which improves the operation efficiency and market competitiveness of the enterprise and at the same time improves the enthusiasm and work efficiency of employees.

## 2. Optimization Analysis Model of Human Resource Allocation in Colleges and Universities Based on Internet of Things Technology

### 2.1. Hierarchical Structure of IoT Technology

**Figure 1:**
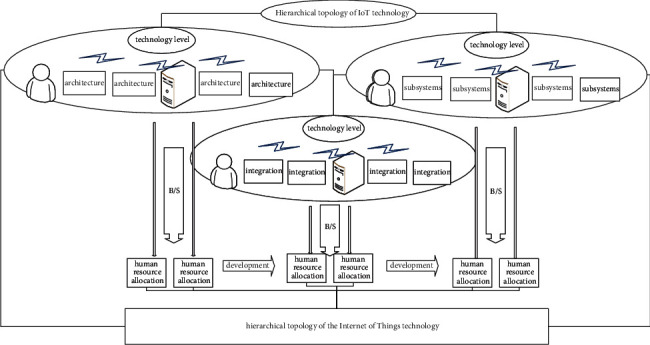
Hierarchical topology of IoT technology.

At the technology level of the Internet of Things, the B/S model is a relatively popular software architecture. It is also a major trend in developing the Internet of Things architecture for large-scale systems in the future. In the B/S mode, users only need to install a browser to directly access the server and exchange data [22–25]. The three-tier system structure of B/S includes a presentation layer, a logical business layer, and a data layer.  Figure 1 is the hierarchical topology of the IoT technology.

In the B/S IoT architecture, this article mainly adopts an open-source Internet of Things architecture that is relatively mainstream, relatively advanced in technology, and relatively mature and stable developed products. In other words, the SSH Internet of Things architecture is used to design and develop. According to the different needs of the system, this paper encapsulates the different subsystems, respectively. The advantage of this is that it is conducive to the integration of the various subsystems after the system development is completed:

(1) $$\begin{array}{c} f_{11}=\left(\frac{1}{n}\times \sum_{i=1}^{n}x_{i}^{2},\frac{1}{n}\times \sum_{i=1}^{n}y_{i}^{2}\right),\\ c=\mathrm{Pr}\left(c\;|\;o\right)\times \mathrm{Pr}\left(o\right)\times \text{IoU}=\mathrm{Pr}\left(c\right)\times \text{Iou}. \end{array}$$

The technology Internet of Things architecture adopted by J2EE mainly adopts the idea of low-coupling hierarchical design and the distributed system Internet of Things architecture, encapsulating different logical structures into different application services for processing, and fully embodies the hierarchical design idea of the system. The presentation layer adopts the framework for design, the business layer mainly adopts the framework, and the data persistence layer adopts the Hibernate framework, which guarantees the system's stability as a whole. In the system development, the Spring structure, Struts structure, and Hibernate framework have become the main flow frameworks for developing a lightweight framework system:

(2) $$\begin{array}{c} l=\alpha \times \sum_{i=0}^{S^{2}}\sum_{j=0}^{B}\left[{\left(x_{i}-\bar{x}\right)}^{2}+{\left(y_{i}-\bar{y}\right)}^{2}+{\left(x_{j}-\bar{x}\right)}^{2}+{\left(y_{j}-\bar{y}\right)}^{2}\right],\\ \psi \left(x\right)=\frac{f\left(x,d\right)-f\left(x,m\right)}{f\left(x,d\right)+f\left(m,d\right)}. \end{array}$$

It can serve the functions of heterogeneous resources, provide services to the outside world with a unified and universal interface, and connect to the manufacturing platform in a loosely coupled manner to realize the full interconnection of the manufacturing services of physical manufacturing resources. However, the current manufacturing resource model mainly models the processing capability of the resource and less considers the real-time status and location of the resource, making it difficult to dynamically optimize based on the real-time status of the manufacturing resource:

(3) $$\begin{array}{c} \chi \left(x\right)=\left\{\begin{array}{cc} e^{-\lambda L\times \left(1+\lambda x^{2}\right)}, & \chi \geq \chi \left(x\right),\\ 0, & \chi <\chi \left(x\right), \end{array}\right.\\ G\left(x\right)=\left\{\begin{array}{c} g\left(x\right)\times \left[0,x,1\right],\;x\subset X,\\ g\left(x\right)\times \left[x,1,0\right],\;x\subset Y,\\ g\left(x\right)\times \left[1,0,x\right],\;x\subset Z. \end{array}\right. \end{array}$$

The real-time manufacturing information acquisition method is the premise and foundation for realizing the service-oriented modeling of dynamic manufacturing resources. According to its information structure, it is hierarchical, structurally related, and gradually superimposed and merged. It can form the basic information of the production process that has practical meaning for manufacturing resources. Then, the information is sorted and combined to form closely related production tasks and production processes. Real-time multisource manufacturing data provide data support for the production information system.

### 2.2. Human Resource Allocation Algorithm

Such an Internet of Things architecture has relatively unified specifications and standards on system components, technical levels, and various service Internet of Things architectures. The advantage of this is that, for different platforms that also follow the J2EE Internet of Things architecture, that is, between heterogeneous platforms, the system performs well in terms of compatibility, allowing the enterprise to communicate well internally and externally. After SPSS software completes logistic regression analysis, the output digital matrix can also be converted into raster graphics in GIS software, thus solving the problem of mutual import of data in GIS and professional statistical analysis software. In the framework of J2EE technology, programmers only need to develop large-scale enterprise-level application systems according to the corresponding specifications, without considering the underlying hardware issues. Considering the compatibility issues between different J2EE platforms and products, different J2EE providers will also support the corresponding standards drawn up in different J2EE versions. In order to facilitate the definition and the expression of operators, this article gives a zero-centered, odd-numbered language term set Hs = {*h*3 = excellent, *h*2 = very good, *h*1 = good, *h*0 = fair, *h*−1 = bad, *h*−2 = very bad, *h*−3 = very bad}. In order to avoid the loss of decision information in language term calculations, language terms are generally expanded. For the language term set of this article, this article adopts the following expansion scale, where *q* is a sufficiently large natural number.

Table 1 is the description of human resource allocation. This layer communicates directly with the database storing the data and can complete the functions of inserting, deleting, modifying, and querying data. When the business logic layer issues a request to the database, the layer responds accordingly. The function of this layer is mainly composed of two functions of OR mapping and database operation layer. It adopts the Hibernate Internet of Things architecture and is based on the OR Mapping framework to carry out persistent package management on the data operation of the system and realize the data and business data of the database. The use of such a framework does not need to consider which database is used, which can effectively ensure the independence of the system. The most resource-consuming system is access to the database operation. Therefore, to improve the content and response performance of the data, the operation of the data access layer on the database must be optimized. The application and realization of the ontology concept in the Web require a standard, common, and standardized language that can be used by computers to describe services. It uses ontology concepts to express the semantic information of Web services and accurately describes the meaning of concepts in a way that can be understood by computers to realize service discovery.

### 2.3. Model Optimization Analysis

Separating the data model of the Internet of Things from the code of the user interface is the main purpose of applying the MVC pattern. According to the MVC Internet of Things architecture model, the application is separated from input, application processing, and output through high-level interfaces between objects to ensure the decoupling between the various parts of the application system and, at the same time, make each layer of the system relatively independent of processing its own business. Its main components include BeanTags, HTMLTags, Logic Tags, NestedTags, and Template Tags. The model is mainly responsible for identifying the state of the system. In this case, the method of using a stepwise linear statistical model will cause large errors in the results and even unreasonable results. As far as the structure of the entire system is concerned, ActionFormBean is used to represent a nonpersistent state. For some persistent states, the Utitle package is used, which is included in Struts itself. The advantage of this is that it becomes more convenient when operating with the database. In the Struts framework, ActionServlet plays the role of the controller, but it is not completely involved in business logic and requires interaction between Action, ActionMapping, and ActionForward. The roles and tasks of the three components are different. The main responsibility for the realization of business logic is action. The difference of business logic is represented by ActionMapping, which is to distinguish. The direction of the process is represented by ActionForward.

**Figure 2:**
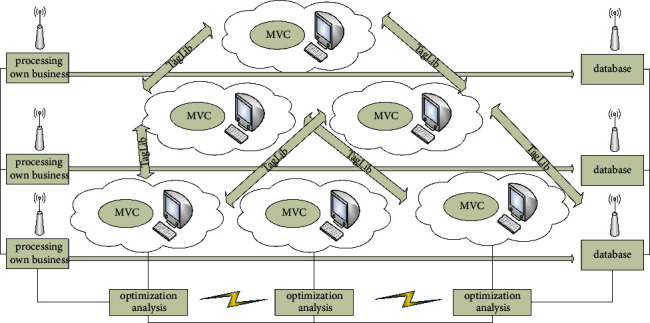
Logic framework of model optimization analysis.

Figure 2 is the logical framework of model optimization analysis. In the manufacturing process, when the sensor node perceives the corresponding event (data), the designed event-driven message transmission mechanism firstly describes the event information collected by the sensor node on the resource side as having {ID, Event, Data, Time} a collection of data structures. Then, the mapping relationship F between different types of sensors and different types of manufacturing information is established in the background. Each sensor event is monitored through multithreading technology, combined with the event-driven IoT architecture (EDA) and mapping table to encapsulate and transmit the data captured by the event on the sensor. The static attributes of the manufacturing resource service give a unique identifier to the service, which summarizes the service capabilities. When a manufacturing task requires a query service, if the query content is a keyword, you can directly query through the UDDI registration center to obtain the universally unique identifier (UUID) of the manufacturing resource service that meets the requirements and return it to the information system. The matching engine finds similar concepts in the ontology database according to the semantic description, calculates the matching degree, obtains the manufacturing resource service that meets the requirements, and finds the UUID of the service in the UDDI registry and returns it to the information system.

### 2.4. Data Weighting Factor Recursion

**Figure 3:**
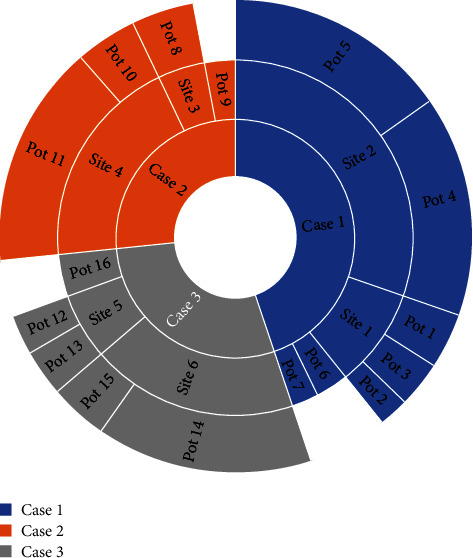
Classification of data weighting factors.

The human resource deployment data Web is an extension of traditional Web services. The resource information in the Semantic Web is no longer a simple information location, but a real way to understand the meaning of information, improve the search, and get high efficiency and intelligence of computers. For the meaning of information on Web computers, the relevant domain knowledge and relationships represented by the information need to be clearly defined. Not only can ontology provide clear definitions of related domain knowledge and mutual relations, but also the logical reasoning capabilities it supports are more conducive to computer understanding and processing. Therefore, the ontology concept model has become the foundation and core of the development of Semantic Web technology. The performance appraisal data comes from the performance appraisal scoring table and relevant system standard benchmarking and original work records. The level of work completion is determined according to the completion of the performance appraisal indicators and is divided into four levels. The actual completion of the work becomes the actual completion of the responsibility objectives and work tasks during the assessment period. The actual assessment scores are scored by the assessment unit (assessor) based on the employee's responsibility target and the completion value of the work task.  Figure 3 shows the classification of data weighting factors.

Through the information collected by the Internet of Things, this article should take an employee by affecting task assignment. When these elements are available, it is not necessary to assign tasks only based on distance. Reasonably, in addition, the Internet of Things will collect data for the frequency distribution, and processing time distribution of emergency resource allocation events in each area can be obtained. We form an effective and comprehensive database, analyze and summarize the data in the database, and slowly find the human resources law from the data, which can provide a scientific basis for future human resources work.

## 3. Results and Analysis

### 3.1. Analysis of IoT Data Characteristics

In order to reflect the status quo of H company's human resource value chain, this paper uses Schuster's human resource index method to conduct a questionnaire survey on H company. The scale used in this method is designed with 64 questions and 15 dimensions. The dimensions, respectively, reflect the main and status quo of the four links of the human resource value chain management model, the relationship between the 15 dimensions of the human resource index, and the 4 dimensions of the human resource value chain, so that the human resource index survey can reflect the management status of the company's human resource value chain. The results show that the proportion of disasters in the 1 and 2 divisions representing low levels of risk has decreased significantly, while the proportion of disasters in the 4 and 5 divisions representing high levels of risk has decreased. The questionnaire adopts the Schechter 5-point scale model, which enables the respondent to answer quickly and conveniently. A total of 112 questionnaires were issued to H company, 105 valid questionnaires were received, and the effective recovery rate was 93.3%. There are 64 questions in the questionnaire, divided into 15 dimensions, and scored using a 5-point scale. The reliability coefficient (Alpha) of this scale is between 0.757 and 0.929.

**Figure 4:**
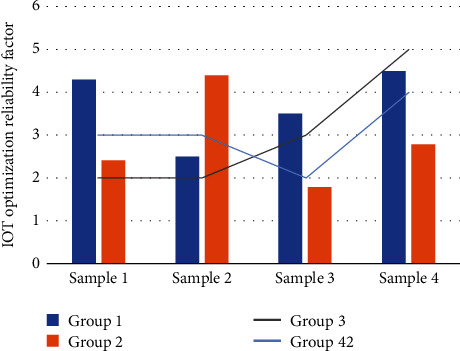
The statistical distribution of the reliability coefficient of the Internet of Things optimization.

Figure 4 is the statistical distribution of the reliability coefficient of the Internet of Things optimization. It can be seen that the scores of each latitude of the H company's human resource index are generally low. Among them, the highest score is the organizational environment 3.45, indicating that the organizational environment of the employees of H company is relatively harmonious. The second highest score is organizational goal 3.25 and group collaboration, indicating that the employees of H company have done a relatively good job in improving information communication, group collaboration, and so on. The lowest score is the remuneration system, with a score of 2.58, indicating that H company's human resources compensation management is lacking, which leads to low employee satisfaction; the second-lowest score is the management quality and the overall management quality of H company is not high. In terms of recruiting and training new recruits, H company lacks reasonable measures and talent mining and value creation. The company does not conduct an in-depth analysis of the company's positions and lacks direction in the company's strategic planning. At the same time, it also reflects that the value evaluation link in the H company's human resource value chain management is not well done, and a new understanding and improvement of the overall management of the organization is needed.

### 3.2. Simulation of Human Resource Allocation

The company expanded related business and elected the new executive director from the four main persons in charge. This article assumes that these four persons are A, B, C, and D. The current work mainly evaluates these four persons. The leaders, colleagues, and subordinates of these four people are evaluated in language terms, and the leaders, colleagues, and subordinates are used as representative groups to participate in the evaluation. The evaluation value of each group selects the group's evaluation range for the person in charge. Based on these theories, the four main persons in charge from the three levels finally obtained the following evaluation information based on the uncertainty language evaluation scale. The city is divided into several large districts, and each district is divided into several blocks. Each block is assigned information collectors and personnel. Therefore, the simulation system is simulated according to the district, and the simulation system is mainly divided into three modules. The first part is the data initialization module. Before the simulation experiment, you need to input the corresponding parameters. The second part is the simulation experiment algorithm part, which is divided into a scheduling module and a configuration module, used to solve the scheduling and configuration problems in the current process and ensure that the simulation experiment normally runs according to the designed logic. The third part is the data interaction module, which interacts with the database, writes necessary data, and reads the required information for queries.  Table 2 is the data interaction module of human resource deployment.

**Figure 5:**
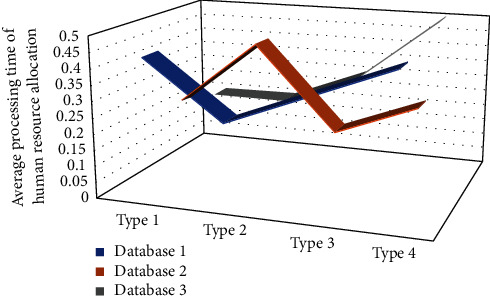
Average processing time of human resource allocation.

Using the ULHA operator in the evaluation of human resources, compared with not using the ULHA operator, the evaluation is more accurate and scientific. It has been used in many human resource management systems and has achieved very good results. The calculation accuracy is more than 10% higher than the traditional method, and this mode is simple to operate, suitable for general scenarios. First of all, the overall index *M* and the change trend of the employee load rate are the same as the previous part. We use the performance appraisal system of the cloud platform to improve the performance management efficiency of the enterprise; the value evaluation process is to check the implementation results of the above two links in the value chain. Salary management is particularly important. Salary management under the cloud platform makes employees more challenging and fair. The final value distribution process has an incentive effect on the entire value chain and is the basis for retaining valuable employees, as shown in Figure 5.

Through the establishment of H company's exclusive HR-cloud system, the cloud system is deployed on the Internet and positioned as a full-staff service, giving full play to the real value of the human resources value chain system and realizing the line of core modules such as personnel, salary, attendance, and performance. At the same time, it can realize employee self-service business processing, full participation in performance management, self-service reporting of training needs, and so on so that the human resources business can truly provide services for senior executives, line managers, and employees. We integrate the human resources of H company and optimize the allocation of company's H human capital as a whole. Through the HR-cloud human resources value chain management platform, employees can timely understand the human resources policies and regulations formulated by the upper leaders of H company; it can supervise the daily human resource management business of various departments to ensure the management system, which greatly improves the satisfaction of employees.

### 3.3. Analysis of Experimental Results

**Figure 6:**
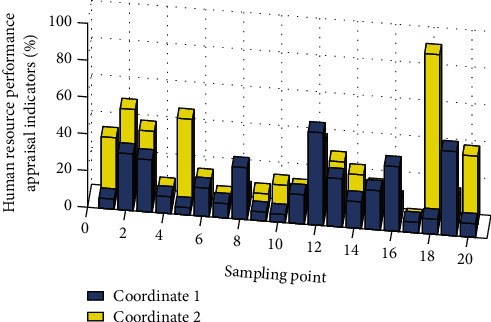
Histogram of human resource performance appraisal indicators.

The human resources performance appraisal indicators are uniformly set as follows: the numbers of performance appraisal indicators for the three types of management personnel of middle management, supervisor, and management assistant are set to 10, respectively; the number of performance appraisal indicators for technical personnel is set to 12; and the numbers of performance appraisal indicators for senior level, standard level, and junior/other three types of production and operation service personnel are set to 10, respectively. The performance evaluation indicators of various types of personnel are divided into three categories: work performance, work behavior, and workability. Each indicator clarifies the specific evaluation content and items. Randomly select the local area model unit, and use the CF value of each factor data type as the independent variable of the logistic regression model and the risk as the dependent variable. Import the data into the SPSS statistical analysis software, and use binary logistic regression analysis. The evaluation weights and standard scores are determined according to the job analysis and job evaluation results, and the evaluation is specified. In the future, according to the company's overall development requirements, performance management needs, and employee development needs, the employee performance appraisal indicator system will be adjusted in a timely manner. The hierarchical overall analysis is used to calculate the weight of the bottom element to the target layer element, that is, the weight of the delivery distance, the priority of the delivery task, and the utilization of the delivery resource for the optimal task plan. The largest eigenvalue of the judgment matrix G1 is an important parameter for consistency testing. The largest eigenvalue corresponding to the eigenvector (orthogonal normalization vector) is called the weight vector. Each component corresponds to the delivery time, the priority of the delivery task, and the resource.  Figure 6 is a histogram of human resource performance appraisal indicators.

Based on the data accumulated by the development of network services, through data processing and analysis, real-time dynamic monitoring of operating conditions collect the dynamic change data of the online public human resources market, implement analysis, display the operation of the online public human resources market, and provide dynamic supervision functions. Due to the lack of opportunities for employees of H company to participate in management, the organizational structure of the Internet industry tends to be flat, with low management personnel, and employees rarely participate in management. However, to realize the potential of employees, it is possible to appropriately increase the opportunities for employees to participate in the management and explore technical skills. In this regard, H company urgently needs improvement in this regard.

**Figure 7:**
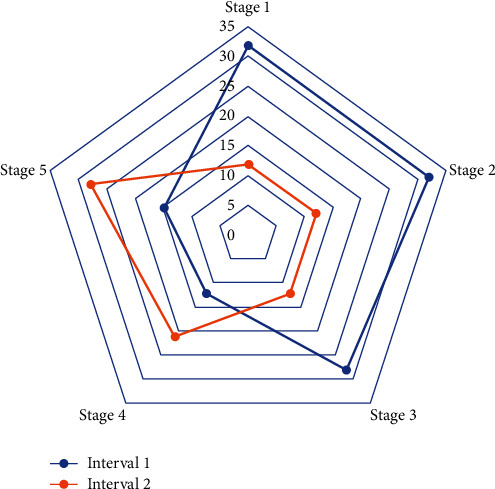
Distribution of real-time dynamic supervision and operation of the Internet of Things.

Figure 7 shows the distribution of real-time dynamic supervision and operation of the Internet of Things. In order to verify the research on the optimization of dynamic resource distribution tasks in the manufacturing Internet of Things environment, this section designs the following manufacturing workshop material distribution scenarios. The proportion of excellent and competent employees reaches 53%. These personnel have good job competence and promotion potential and can be used as future talent reserves. Incompetent personnel belong to the department of the R&D center. The out-buffer is used to store materials that have been processed by the workstation and used in other manufacturing links; the second is the storage area, which contains a series of three-dimensional shelves to store and maintain the normal workshop; and the third is a forklift, which has real-time perception service capabilities by configuring sensors and RFID and other equipment. At the same time, relevant hardware devices are also attached to key locations and resource storage areas to collect real-time distribution information, such as attaching RFID tags at key intersections, material buffers, and shelves in the workshop to collect location information.

## 4. Conclusion

This system mainly uses the B/S Internet of Things architecture for design and development. First, it can effectively protect the security of sensitive data, especially when adding and modifying records to the database. It can make general functions easier to use and ensure that complex functions interact. At the same time, it focuses on the problem of service modeling based on real-time information, service combination optimization, and resource distribution optimization of dynamic manufacturing resources in the manufacturing Internet of Things environment. Under the guidance of the resource-as-a-service ideology, a manufacturing resource modeling system with real-time perception service capabilities is organized, and the manufacturing resource contextual information modeling method, manufacturing resource servicing modeling method, and manufacturing resource service combination tasks are detailed respectively. This module uses a histogram to analyze the comparison between the existing emergency source capacity of the district and the optimized configuration. The modeling method establishes the research foundation for the dynamic manufacturing resource service combination optimization and resource distribution optimization later. In the manufacturing Internet of Things workshop, human resources, material resources, and equipment resources are the main research objects, a context-aware manufacturing resource service discovery mechanism is designed, a service composition model based on workflow diagrams is established, and gray correlation is adopted. The proposed human resource allocation plan simulation system uses the historical data collected in the smart city to deal with emergency human resources problems in various regions for simulation experiments, which can help city managers find the plan for each region based on their own needs to deal with emergency human resources issues.

## Figures and Tables

**Table 1:** Description of human resource allocation.

Index	Dataflow rate (%)	Expansion scale	Factor weight
1	17	1.21	0.38
2	14	2.01	0.09
3	25	1.89	0.12
4	31	1.77	0.41

**Table 2:** Data interaction module for human resource deployment.

Number	Description	Value
1	Type, resource, quality, volume	20
2	Model, load capacity, module volume, transportation distance	40
3	Distribution demand, distribution frequency, distribution point	20
4	Module resource distance, number of modules used, delivery time limit	40
5	Meet time resource requirements, meet customer distribution needs	50
6	Distribution center to customer path, customer to customer path	70
7	Gathering goods, completion of module distribution tasks	60

## Data Availability

The data used to support the findings of this study are available from the corresponding author upon request.
